# Interpretation of cytogenetic results in multiple myeloma for clinical practice

**DOI:** 10.1038/bcj.2015.92

**Published:** 2015-10-30

**Authors:** A M Rajan, S V Rajkumar

**Affiliations:** 1 Aureus University School of Medicine, Oranjestad, Aruba; 2 Division of Hematology, Mayo Clinic, Rochester, MN, USA

## Abstract

The interpretation of cytogenetic abnormalities in multiple myeloma (MM) is often a challenging task. MM is characterized by several cytogenetic abnormalities that occur at various time points in the disease course. The interpretation of cytogenetic results in MM is complicated by the number and complexity of the abnormalities, the methods used to detect them and the disease stage at which they are detected. Specific cytogenetic abnormalities affect clinical presentation, progression of smoldering multiple myeloma (SMM) to MM, prognosis of MM and management strategies. The goal of this paper is to provide a review of how MM is classified into specific subtypes based on primary cytogenetic abnormalities and to provide a concise overview of how to interpret cytogenetic abnormalities based on the disease stage to aid clinical practice and patient management.

## Introduction

Multiple myeloma (MM) is a cytogenetically heterogenous plasma cell malignancy.^1, 2, 3^ Several recurrent cytogenetic abnormalities are seen throughout the course of the disease, from the premalignant stage of monoclonal gammopathy of undetermined significance (MGUS) to smoldering multiple myeloma (SMM) to end-stage MM.^4, 5^ Some abnormalities start at the time of initial transformation of a normal plasma cell to the limited clonal proliferative state of MGUS, while some occur later in the disease course as the malignancy progresses to a more relapsed refractory state.^6, 7, 8^ Cytogenetic abnormalities in MM affect every aspect of the disease, from evolution of the malignancy to clinical presentation, response to therapy and prognosis. A given abnormality may have a totally different meaning based on the disease stage. For example, trisomies are associated with a higher risk of transformation from SMM to MM but lower risk of progression from onset of MM to end-stage disease.^9, 10, 11^ The sheer number and complexity of cytogenetic abnormalities that occur in MM and the multiple ways in which each can affect patient care and counseling make the evaluation and interpretation of cytogenetic abnormalities in MM a daunting task. The purpose of this review is to provide a concise and succinct overview of the interpretation of cytogenetic results in MM that is directly relevant to clinical practice.

The goal of this paper is not to review the underlying biological or pathogenetic mechanisms but rather to assist the clinician in patient management. The main areas of focus will be classification of MM into cytogenetically distinct subtypes, laboratory testing strategy for practice, prediction of progression in SMM, influence of cytogenetics on disease presentation, risk stratification and prognosis of MM and selection of therapy.

## Nature of cytogenetic abnormalities and classification of MM

**Figure 1:**
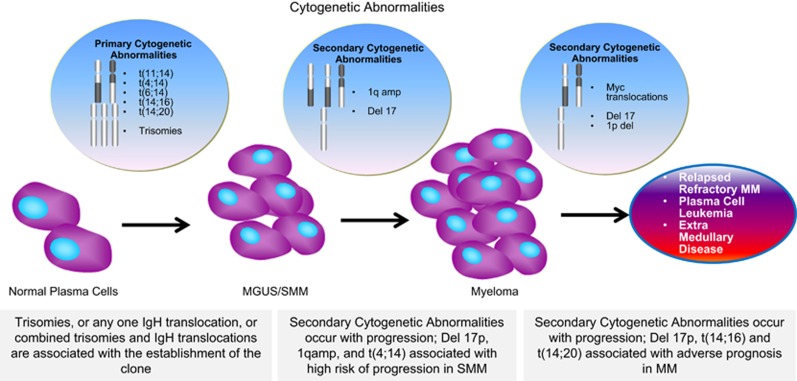
Cytogenetic abnormalities in multiple myeloma. Primary cytogenetic abnormalities occur early when the normal plasma cell transitions to a clonal, premalignant stage. Most secondary cytogenetic abnormalities occur later in the disease course with malignant transformation or during progression of the malignancy. The effect of primary and secondary cytogenetic abnormalities on prognosis depends on the disease.

There are two broad types of cytogenetic abnormalities in MM: primary and secondary. Primary cytogenetic abnormalities classify MGUS and MM into several distinct, mostly non-overlapping subtypes (Table 1).^6^ They are thought to occur at the time of MGUS and are believed to have a role in the initial pathogenesis of MGUS. Secondary cytogenetic abnormalities can occur in any of the primary subtypes of MM and influence disease outcome to varying degrees. In contrast to primary cytogenetic abnormalities, they are overlapping and many different secondary cytogenetic abnormalities can occur in the same patient (Figure 1).

### Primary cytogenetic abnormalities

These abnormalities essentially classify MM into several subtypes.^11, 12^ In fact, it is likely each represents a unique cytogenetically distinct disease (Table 1).^5, 12, 13, 14^ There are two main types of primary cytogenetic abnormalities in MM: trisomies and translocations involving the immunoglobulin heavy chain (IgH) gene. The trisomic form of MM is characterized by an extra copy of one or more odd-numbered chromosomes (chromosomes 3, 5, 7, 9, 11, 15, 17). The IgH-translocated form of MM includes several distinct subtypes, the most common being t(11;14), t(4;14), t(6;14), t(14;16) and t(14;20).^15^ In each of these translocations, an oncogene from a partner chromosome is translocated to the IgH region on chromosome 14q32. Thus the genes dysregulated in these translocations are: 11q13 (CCND1 (cyclin D1 gene)), 4p16.3 (FGFR-3 and MMSET), 6p21 (CCND3 (cyclin D3 gene)), 16q23 (c-MAF), and 20q11 (MAF-B), respectively.^16, 17, 18^ A small subset of patients with MM has evidence of both trisomies and IgH translocations, but in general, the primary cytogenetic subtypes are considered to be non-overlapping. Thus a given patient with MM will not have both t(11;14) and t(4;14). Occasional patients with MM may lack both IgH translocations and trisomies but have isolated monosomy 14, translocations involving the immunoglobulin light chain loci on chromosomes 2 or 22 or other abnormalities. It is possible that failure to detect trisomies or IgH translocations in this group of patients may be related to technical reasons such as the lack of the appropriate probes for fluorescent *in situ* hybridization (FISH). Some patients with MM have no detectable cytogenetic abnormalities; most of the time, this is due to insufficient plasma cells for analysis, while in others it likely reflects the fact that cells have a rare abnormality that is not targeted by the probes used for detection.

### Secondary cytogenetic abnormalities

Numerous secondary cytogenetic abnormalities have been described in MM. One or more of these can occur in any of the primary cytogenetic subtypes of MM. One of the earliest described secondary cytogenetic abnormality is monosomy 13 or del(13q), initially detected in metaphase cytogenetic studies.^19^ Early studies showed that monosomy 13/del(13q) was a significant adverse prognostic marker in MM, but later studies showed the prognostic effect was primarily seen when the abnormality was detected by conventional karyotypic (rather than FISH) studies, where the abnormality probably functions as a surrogate marker for hypodiploidy, IgH translocations or proliferation rather than being a true driver of risk.^19, 20, 21, 22^ Monosomy 13/del(13q) is an early event in MM pathogenesis and is seen in up to 50% of patients with MGUS.^18^ The frequent occurrence of monosomy 13/del(13q) in MGUS and SMM indicates the need for further study of this abnormality regardless of the lack of a true prognostic effect.

Deletions involving chromosome 17p, referred to as del(17p), typically occur later in the disease course. The finding of del(17p) or monosomy 17 in SMM indicates a high risk for progression to MM,^9, 10^ while its detection in a patient with newly diagnosed or relapsed MM indicates an adverse prognosis for progression-free survival (PFS) and overall survival (OS).^5, 12, 23, 24, 25^

A duplication of chromosome 1q21, referred to as gain(1q21), has been noted in >40% of patients with SMM and MM compared with 0% in MGUS,^26^ suggesting that gain(1q21) may have a role in disease progression.^27^ In fact, studies show that gain(1q21) is associated with a higher risk of progression in SMM and unfavorable outcome in MM.^10, 23, 25^

Other secondary cytogenetic abnormalities of clinical interest include *MYC* translocations and del(1p), both of which has been associated with adverse prognosis in MM.^28, 29, 30, 31^

## Laboratory testing strategy

For clinical purposes, cytogenetics in MM can be assessed by metaphase karyotyping (conventional cytogenetics) or by FISH. Metaphase cytogenetics requires proliferating cells and is not sensitive for the detection of either primary or secondary cytogenetic abnormalities in MM. Further, any prognostic impact that is seen with a metaphase-detected abnormality is probably not due to that abnormality *per se* but simply a reflection of the fact that the patient has a more proliferative, aggressive form of MM. Thus, in general, metaphase cytogenetics are mainly useful to determine the presence of myelodysplastic syndrome that may occur during the course of the disease secondary to therapy.

FISH examination in MM is carried out in conjunction with staining for cytoplasmic immunoglobulin. This allows us to determine whether the abnormality is present in the plasma cell clone or other hematopoietic cells. A general FISH analysis for MM should ideally include fluorescent probes to detect trisomies, IgH translocations, *MYC* translocations and abnormalities of chromosomes 1, 13 and 17. The procedures used for cytoplasmic immunoglobulin–FISH studies at the Mayo Clinic, Rochester, MN, USA have been previously described.^18, 22^ The probes used at Mayo Clinic for newly diagnosed MM are: 1p36.3(TP73), 1q21(gain), 3cen (D3Z1), 7cen (D7Z1), 8q24 (3'MYC,5'MYC), 9cen (D9Z1), 15cen (D15Z4), 11q13 (CCND1-XT), 13q14 (RB1), 13q34 (LAMP1), 14q32 (IGH-XT), 14q32 (5'IGH,3'IGH), 17p13.1 (p53), and 17cen (D17Z1). Most abnormalities of chromosome 13 represent monosomy 13, while a small percentage are del(13q); in contrast, most abnormalities of chromosome 17 are del(17p) and only a small percentage are monosomy 17. Additional probes as needed are used to detect t(4;14), t(6;14), t(14;16), t(14;20) and other abnormalities based on the results of the initial screen.

A comprehensive FISH probe set as described above is only needed once for MM. Once the primary cytogenetic subtype of MM is identified, with repeat bone marrow examinations carried out at relapse a more limited probe set is adequate, for example: 1p36.3(TP73), 1q21(CKS1B), 8q24 (3'MYC,5'MYC), 17p13.1 (p53), and 17cen (D17Z1).

Other more advanced methods such as RNA sequencing, comparative genomic hybridization or whole-genome sequencing are not yet commonly used in clinical practice.

In general, a patient can be classified into specific molecular classification subtypes of MM as shown in Table 1, regardless of when in the disease course these abnormalities are detected as they are present from the initial MGUS stage. Conversely, when a secondary cytogenetic abnormality such as gain(1q21) or del(17p) is detected one cannot ascertain when the abnormality first appeared unless sequential results are available.

## Primary cytogenetic abnormalities and clinical presentation of MM

As discussed in the previous section, MM can be classified into multiple subtypes based on the underlying primary cytogenetic subtype. The fact that prognosis of MM varies across the primary cytogenetic subtypes is well known. Studies show that the clinical presentation of MM is also influenced to some degree by the underlying cytogenetic subtype. In a study conducted at the Mayo Clinic, 484 MM patients were classified based on the primary cytogenetic subtype, and the clinical and laboratory features of this cohort were examined in detail.^32^ The study found several important associations of clinical significance. First, MM with IgH translocations was more commonly associated with high free light chain levels and renal failure as the myeloma-defining event (MDE). Specifically, t(14;16) MM accounted for only 5% of study cohort but was seen in 14% of patients with renal failure as the MDE. In fact, 25% of patients with t(14;16) MM presented with renal failure only as the initial MDE. Second, patients with t(11;14) and t(6;14) MM tended to present more often with bone disease as the initial MDE compared with patients who had t(4;14) or t(14;16). Third, in contrast to differences in occurrence of renal failure and bone disease based on the underlying primary cytogenetic subtype, no differences in the occurrence of anemia as MDE was noted across the cytogenetic subtypes.

## Predicting risk of progression of SMM

Although numerous studies have described the role of cytogenetic abnormalities in prognosis of MM,^5, 12, 33, 34^ only a few studies have examined the influence of cytogenetic abnormalities on the risk of progression of SMM to MM.

In a Mayo Clinic study of 351 patients with SMM, 154 patients (43.9%) had trisomies, 127 (36.2%) had IgH translocations, 14 (4%) both trisomies and IgH translocations, 53 (15.1%) no abnormalities detected and 3 (0.9%) had monosomy 13/del(13q) in the absence of any other abnormality.^9^ During the follow-up period, 219 patients with SMM (62.4%) progressed to MM. Time to progression of SMM to MM could be risk stratified in a clinically meaningful way based on the underlying cytogenetic abnormality: t(4;14) and del 17p abnormalities (high-risk SMM), trisomies alone (intermediate risk), t(11;14), or other IgH translocations (standard-risk SMM), and no detectable abnormalities (low risk) (Table 2). Monosomy 13/del(13q) had no impact on risk of progression.

Similar results have also been reported by Neben *et al.*^10^ in a study of 248 patients with SMM. Del(17p13), t(4;14) and gain(1q21) were present in 6, 9 and 30% of patients with SMM, respectively. All were associated with a higher risk of progression to MM. Trisomies were also associated with a higher risk of progression.

The high risk of progression of SMM to MM with t(4;14) may be related to the fact that this abnormality is associated with markedly high free light chain ratios.^35^ However, the mechanism by which a high free light chain ratio is associated with higher risk of progression is not clear and is only partly related to renal failure from cast nephropathy.

SMM patients with t(4;14) translocation, del(17p) and gain(1q21) should be considered as having high-risk SMM.^9, 10, 36^ They should be offered clinical trials testing early intervention. They also need close follow-up indefinitely every 3–4 months.^37^ Recent data favor early intervention with lenalidomide–low-dose dexamethasone (Rd) in high-risk patients.^38^ Therefore, if SMM patients with t(4;14) translocation, del(17p), and gain(1q21) have multiple other adverse prognostic factors for progression, consideration can be given for preventive therapy.^36^

## Prognosis and risk stratification of MM

The detection and interpretation of cytogenetic abnormalities in MM is of critical importance for prognosis and risk stratification of MM (Table 3).^39^ In one study, patients with MM without t(4;14), del(17p) or gain(1q21) who had stage I or II MM by the International Staging System (representing approximately 20% of all patients with MM) had an 8-year survival of 75%.^40^ In contrast, studies show that patients with high-risk cytogenetics (Table 3) have a median OS of <2–3 years despite best available treatments and are candidates for innovative, more aggressive clinical trials.^14^ It must be emphasized that most data that support the use of cytogenetic abnormalities for prognosis and risk stratification are based on studies carried out in patients with newly diagnosed MM. However, many are of value in relapsed refractory MM as well.^34^ At the Mayo Clinic, these abnormalities are used to classify both newly diagnosed MM and relapsed MM into standard-, intermediate- and high-risk disease using the Mayo stratification for myeloma and risk-adapted therapy classification (mSMART) (www.msmart.org).^15, 41^

Patients are considered to have high-risk disease if FISH studies demonstrate one of the following abnormalities: t(14;16), t(14;20), or loss of p53 gene locus (del(17p) or monosomy 17).^23, 39^ There is some controversy about whether there is a true adverse biological risk associated with t(14;16) MM. This form of MM is more frequently associated with acute renal failure as the MDE at diagnosis.^32^ Thus in clinical trials that typically exclude patients with renal failure an adverse prognosis associated with t(14;16) MM may not be seen, in contrast to studies which include all patients seen in a given institution where an unfavorable outcome has been observed.^12^ In one study, after adjusting for renal failure, the outcome of t(14;16) was comparable to other standard-risk subtypes of MM. Recent studies show that del(1p) may also signal high-risk MM. In a study of 1195 patients by the Intergroupe Francophone du Myeloma, del(1p) and specifically del(1p22) and del(1p32) were adverse prognostic factors for both progression-free survival and OS.^42^ OS in this group of patients treated with induction therapy and autologous stem cell transplantation was only 27 months in patients with del(1p32) versus 97 months without, *P*<0.001. In multivariate analyses, del(1p22) and del(1p32) were independent of t(4;14), t(14;16) and del(17p).

As seen in Table 3, the prognostic risk is driven primarily by the underlying primary cytogenetic subtype of MM. However, secondary cytogenetic abnormalities, del(17p), del(1p) and gain(1q21), also influence outcome. The occurrence of gain(1q21) moves standard-risk patients automatically into the intermediate-risk category, while the occurrence of del(17p), and possibly del(1p), indicates high-risk disease regardless of the underlying primary cytogenetic subtype. Trisomies have been shown to ameliorate the effect of high-risk cytogenetic abnormalities^11^ but not all studies have confirmed this finding.^25^ In addition to the abnormalities listed in Table 3, others that may have additional prognostic value in MM include *MYC* translocations.^28^

## Using cytogenetic results to guide therapy

Accurate detection and interpretation not only assists in counseling patients regarding anticipated outcome but also helps in choice of drugs and in selecting overall therapeutic strategy.^8^ Patients with trisomies not only have an excellent outcome overall but also seem to respond particularly well to lenalidomide-based therapy.^43^ Patients with t(4;14) who have traditionally had significantly inferior outcome are now able to have an OS similar to patients with standard-risk MM when treated with bortezomib-containing regimens and autologous stem cell transplantation.^44, 45, 46, 47^ Recent studies show that early use of bortezomib, autologous stem cell transplantation and bortezomib-based maintenance may improve outcome significantly in patients with high-risk MM owing to del(17p).^48^ These examples illustrate how we can use cytogenetic data to provide prognostic information to patients and also use these data to guide management. In the Mayo Clinic mSMART approach, for example, using these data maintenance with bortezomib is preferred for intermediate- and high-risk patients while lenalidomide maintenance is considered for standard-risk patients.^39^

## Practical approach to interpretation

Table 4 provides a summary guide to the interpretation of the most common cytogenetic abnormalities that are encountered by clinicians on bone marrow examination reports. The interpretations provided are based on cytogenetic abnormalities detected on FISH testing. As shown in Table 4, the interpretation of the specific abnormality will be influenced by the disease stage at which the patient is undergoing testing, SMM versus MM. Time to progression and OS estimates assume that the patient is newly diagnosed with SMM or MM.^9, 10, 39^ In patients with relapsed MM, the cytogenetic abnormalities probably carry the same prognostic effect, although the OS estimates will be influenced by how many relapses have occurred, duration of previous remission and the number of available treatment opportunities available in addition to the nature of the cytogenetic abnormalities.^49, 50^ Trisomies, IgH translocations and monosomy 13/del(13q) can be detected on FISH testing in MGUS if sufficient numbers of plasma cells are present in the sample. These abnormalities, as discussed earlier, start in the MGUS stage and are not indicative of malignant transformation. However, there are limited data on whether there are differences in the risk of progression of MGUS based on the type of abnormality detected. However, if del(17p) is observed in a patient with suspected MGUS it may indicate a higher risk for progression or an error in the diagnosis.

Detection of any cytogenetic abnormality on conventional metaphase cytogenetics indicates a more proliferative form of MM and hence an adverse prognosis. The prognostic effect is more due to the very fact that informative cytogenetic results were obtained rather than the specific abnormality detected. Thus the presence of trisomies on metaphase cytogenetic studies do not carry the same good risk implications as they do when detected by FISH. Detection of complex cytogenetic abnormalities (⩾3 abnormalities), hypodiploidy, monosomy 13/del(13q) or monosomy 17/del(17p) on conventional cytogenetics in a patient with MM should be considered as indicative of a more adverse prognosis.^5, 51^ Depending on the abnormality, the finding of cytogenetic abnormalities on conventional metaphase cytogenetics may also indicate the development of secondary myelodysplastic syndrome. If abnormalities of any kind are detected on conventional cytogenetics in a patient with suspected MGUS or SMM, it may indicate a higher risk for progression, an error in the diagnosis or it is possible that the abnormalities are not arising from plasma cells but rather other hematopoietic cell lines and may indicate coexistent disorders, such as myelodysplastic syndrome.

In summary, cytogenetic abnormalities detected on standard FISH testing are of significant value in classification, risk stratification and management of patients with SMM and MM. Cytogenetic assessment of MM is essential for clinical practice, and the importance of this evaluation is indicated by the recent incorporation of high-risk cytogenetic abnormalities into the Revised International Staging System for MM.^52^

## Figures and Tables

**Table 1:** Primary molecular cytogenetic classification of multiple myeloma

*Subtype*	*Gene(s)/chromosomes affected*^a^	*Percentage of myeloma patients*
Trisomic MM	Recurrent trisomies involving odd-numbered chromosomes with the exception of chromosomes 1, 13 and 21	42
		
*IgH-translocated MM*		30
t(11;14) (q13;q32)	*CCND1* (cyclin D1)	15
t(4;14) (p16;q32)	*FGFR-3* and MMSET	6
t(14;16) (q32;q23)	*C-MAF*	4
t(14;20) (q32;q11)	*MAFB*	<1
Other IgH translocations^a^	*CCND3* (cyclin D3) in t(6;14) MM	5
		
Combined IgH-translocated/trisomic MM	Presence of trisomies and any one of the recurrent IgH translocations in the same patient	15
Isolated monosomy 14	Few cases may represent 14q32 translocations involving unknown partner chromosomes	4.5
Other cytogenetic abnormalities in the absence of IgH translocations or trisomy or monosomy 14		5.5
Normal		3

Abbreviations: IgH, immunoglobulin heavy chain; MM, multiple meloma. Modified from Kumar *et al.*^11^

a Includes the t(6;14)(p21;q32) translocation and, rarely, other IgH translocations involving uncommon partner chromosomes.

**Table 2:** Cytogenetic risk stratification of smoldering multiple myeloma

*Risk*	*Cytogenetic abnormalities*	*Percentage of patients (*N*=351)*	*Median TTP to multiple myeloma (months)*^a^	*Median TTP to multiple myeloma or related disorder (months)*^b^	*Median OS from SMM diagnosis (months)*^c^	*Median OS from MM diagnosis (months)*^d^
High risk	t(4;14) Del(17p) Gain(1q21)^e^	13%	24	24	105	60
Intermediate risk	Trisomies	42%	34	34	135	77
Standard risk	Other abnormalities (includes t(11;14), t(14;16), t(14;20)), combined IgH translocations and trisomies^f^ and isolated monosomy 13	30%	55	54	147	86
Low risk	No abnormalities detected on FISH^g^	15%	Not reached	101	135	112

Abbreviations: FISH, fluorescence *in situ* hybridization; IgH, immunoglobulin heavy chain; MM, multiple myeloma; OS, overall survival; SMM, smoldering multiple myeloma; TTP, time to progression.

a *P*=0.001, ^b^*P*=0.002, ^c^*P*=0.12 (global); *P*=0.02 (high risk versus standard risk), ^d^*P*=0.04 Modified from Rajkumar *et al.,*^9^

e gain(1q21) was not part of this study but was included in the Table based on data from Neben *et al.*^10^

f Except t(4;14), which is considered high risk with or without concurrent trisomies.

g Implies adequate probes used to detect del 17p, 1qamp, trisomies and common IgH translocations.

**Table 3:** Cytogenetic risk stratification of myeloma

*Risk stratification*	*Cytogenetic abnormalities*
Standard risk^a^	Trisomies t(11;14) t(6;14)
Intermediate risk^a^	t(4;14) Gain(1q21)
High risk	Del(17p) t(14;16) t(14;20) Del(1p)

Modified from Rajkumar.^1^

a Presence of del 17p indicates high risk MM regardless of other abnormalities; gain(1q21) (without other high risk abnormalities) is considered intermediate-risk.

**Table 4:** Practical Guide to Interpretation of Cytogenetic Abnormalities detected by FISH in Clinical Practice

*Cytogenetic abnormality*	*Clinical setting in which abnormality is detected*	
	*SMM*	*MM*
Trisomies	Intermediate risk of progression, median TTP of 3 years	Good prognosis, standard-risk MM, median OS 7–10 years Most have myeloma bone disease at diagnosis Excellent response to lenalidomide-based therapy
t(11;14) (q13;q32)	Standard risk of progression, median TTP of 5 years	Good prognosis, standard-risk MM, median OS 7–10 years
t(6;14) (p21;q32)	Standard risk of progression, median TTP of 5 years	Good prognosis, standard-risk MM, median OS 7–10 years
t(4;14) (p16;q32)	High risk of progression, median TTP of 2 years	Intermediate-risk MM, median OS 5 years Needs bortezomib-based initial therapy and early ASCT (if eligible), followed by bortezomib-based consolidation/maintenance
t(14;16) (q32;q23)	Standard risk of progression, median TTP of 5 years	High-risk MM, median OS 3 years Associated with high levels of FLC and 25% present with acute renal failure as initial MDE
t(14;20) (q32;q11)	Standard risk of progression, median TTP of 5 years	High-risk MM, median OS 3 years
Gain(1q21)	High risk of progression, median TTP of 2 years	Intermediate-risk MM, median OS 5 years
Del(17p)	High risk of progression, median TTP of 2 years	High-risk MM, median OS 3 years
Trisomies plus any one of the IgH translocations	Standard risk of progression, median TTP of 5 years	May ameliorate adverse prognosis conferred by high-risk IgH translocations and del 17p
Isolated monosomy 13 or isolated monosomy 14	Standard risk of progression, median TTP of 5 years	Effect on prognosis is not clear
Normal	Low risk of progression, median TTP of 7–10 years	Good prognosis, probably reflecting low tumor burden, median OS >7–10 years

Abbreviations: ASCT, autologous stem cell transplantation; FISH, fluorescent *in situ* hybridization; FLC, free light chain; IgH, immunoglobulin heavy chain; MDE, myeloma-defining event; MM, multiple myeloma; OS, overall survival; SMM, smoldering multiple myeloma, TTP, time to progression.
